# Potential of whole-body dual-energy X-ray absorptiometry to predict muscle size of psoas major, gluteus maximus and back muscles

**DOI:** 10.1186/s12891-023-07051-z

**Published:** 2023-11-27

**Authors:** Masaru Tanaka, Masahiro Kanayama, Fumihiro Oha, Yukitoshi Shimamura, Takeru Tsujimoto, Yuichi Hasegawa, Tomoyuki Hashimoto, Hidetoshi Nojiri, Muneaki Ishijima

**Affiliations:** 1 Spine Center, Hakodate Central General Hospital, Hon-cho 33-2, Hakodate, Hokkaido 040-8585 Japan; 2 Department of Medicine for Orthopaedics and Motor Organ, Juntendo University Graduate School of Medicine, 1-5-29-4F, Yushima, Bunkyo-ku, Tokyo, 113-0034 Japan

**Keywords:** Trunk muscle mass, Dual-energy X-ray absorptiometry, Cross-sectional areas, Psoas major, Back muscles, Gluteus Maximus

## Abstract

**Background:**

Measurement of trunk muscle cross-sectional area (CSA) using axial magnetic resonance imaging (MRI) is considered clinically meaningful for understanding several spinal pathologies, such as low back pain and spinal sagittal imbalance. However, it remains unclear whether trunk muscle mass (TMM) measured using dual-energy X-ray absorptiometry (DXA) can predict the trunk muscle CSA. The aim of this study is to determine if DXA-derived TMM is associated and predicts with CSA of paraspinal muscles and gluteus maximus measured using MRI in healthy volunteers.

**Methods:**

A total of 48 healthy volunteers underwent whole-body DXA and MRI of the spinopelvic region. The CSA of the psoas major, back muscles, and gluteus maximus were measured on axial MRI. Correlations and linear regressions between the TMM measured using DXA and the CSA of each musculature were investigated.

**Results:**

There was a weak correlation between TMM and CSA of the psoas major in men (r = 0.39, *P* = 0.0678), and the linear regression was y = 301.74x – 401.24 (R^2^ = 0.2976, *P* = 0.0070). A moderate correlation was found in women (r = 0.58, *P* = 0.0021), and the linear regression was y = 230.21x − 695.29 (R^2^ = 0.4445, *P* = 0.0003). Moderate correlations were observed between TMM and CSA of the back muscles in both men (r = 0.63, *P* = 0.0012) and women (r = 0.63, *P* = 0.0007), the linear regression was y = 468.52x + 3688.5 (R^2^ = 0.5505, *P* < 0.0001) in men and y = 477.39x + 2364.1 (R^2^ = 0.564, *P* < 0.0001) in women. There was a strong correlation between TMM and CSA of the gluteus maximus in men (r = 0.72, *P* < 0.0001), and the linear regression was y = 252.69x − 880.5 (R^2^ = 0.6906, *P* < 0.0001). A moderate correlation was found in women (r = 0.69, *P* < 0.0001), and the linear regression was y = 230.74x – 231.32 (R^2^ = 0.6542, *P* < 0.0001).

**Conclusions:**

The DXA-derived TMM was able to predict the CSA of the psoas major, back muscles, and gluteus maximus, and significantly correlated with the CSA of the back muscles and gluteus maximus. It might be a safer and cheaper alternative for evaluating the size of the back muscles and gluteus maximus.

## Background

Trunk muscles play an important role in supporting the spinal column, and atrophy of trunk muscles, especially the lumbar paraspinal muscles, affects several spinal pathologies [1–6]. Trunk muscle size is generally estimated from the cross-sectional area (CSA) of the lumbar paraspinal muscles on magnetic resonance imaging (MRI) or computed tomography (CT). The relationship between low back pain and the CSA of the lumbar paraspinal muscles has been shown in previous studies [1–3]. Ranger et al. showed that lumbar paraspinal muscle CSA is associated with low back disability [4]. In recent years, it has been reported that the CSA of the paraspinal muscles in the lumbar spine is closely related to adult spinal deformity, which is the main pathology of spinal sagittal imbalance [5, 6]. However, most of these studies were based on MRI or CT assessments, and it is difficult to conduct large-scale research studies because of radiation exposure, time required for the examination, facilities, and costs. Therefore, a simpler and less expensive measurement of the trunk muscle size is of great clinical importance.

DXA is often used in the management of osteoporosis, diagnosing sarcopenia, and studying body composition [7–9]. Previous studies have reported a positive relationship between appendicular MRI-derived muscle CSA and DXA-derived lean mass [10–12]. Moreover, this relationship has also been observed in the axial skeleton in recent studies [13, 14]. Only a few studies have established this relationship in the spine and not in healthy populations; one study was conducted in patients with low back pain [15] and another in patients with spinal cord injury [16]. No studies determine if DXA-derived trunk lean mass predicts axial MRI-derived CSA in the healthy populations. Although measurement of lumbar paraspinal muscle CSA using axial MRI is clinically important for understanding spinal pathologies, it remains unclear whether DXA-derived trunk muscle mass, which is determined by the value of trunk lean mass, which is a lean mass in the body regions excluding head and all appendicular (upper and lower limbs) regions from body composition data can predict the CSA of lumbar paraspinal muscles (psoas major and back muscles) and gluteus maximus. To determine if DXA-derived trunk muscle mass is associated and predicts with CSA of the psoas major, back muscles, and gluteus maximus, we investigated the correlation and linear regression between trunk muscle mass measured using DXA and CSA of the psoas major, back muscles, and gluteus maximus measured using axial MRI of the lumbar spine and pelvis in healthy volunteers.

## Methods

The study participants underwent whole-body DXA and MRI of the lumbar spine and pelvis. It was necessary to obtain data from healthy individuals whose lives were not impaired by low back pain. Individuals with low back pain requiring sick leave and/or under any modality of treatment (medication, physiotherapy, epidural/nerve root injections, candidates for surgeries, etc.) were excluded from the study. Patients who had previously undergone lumbar or hip surgery were also excluded. The Oswestry Disability Index (ODI) and Roland-Morris Disability Questionnaire (RDQ) are two disability questionnaires most used as outcome measures in patients with low back pain [17, 18]. The ODI and RDQ were used to ensure that none of the participants had function-limiting low back pain. None of the participants had an ODI > 15% or an RDQ score > 6 points.

### DXA

**Fig. 1:**
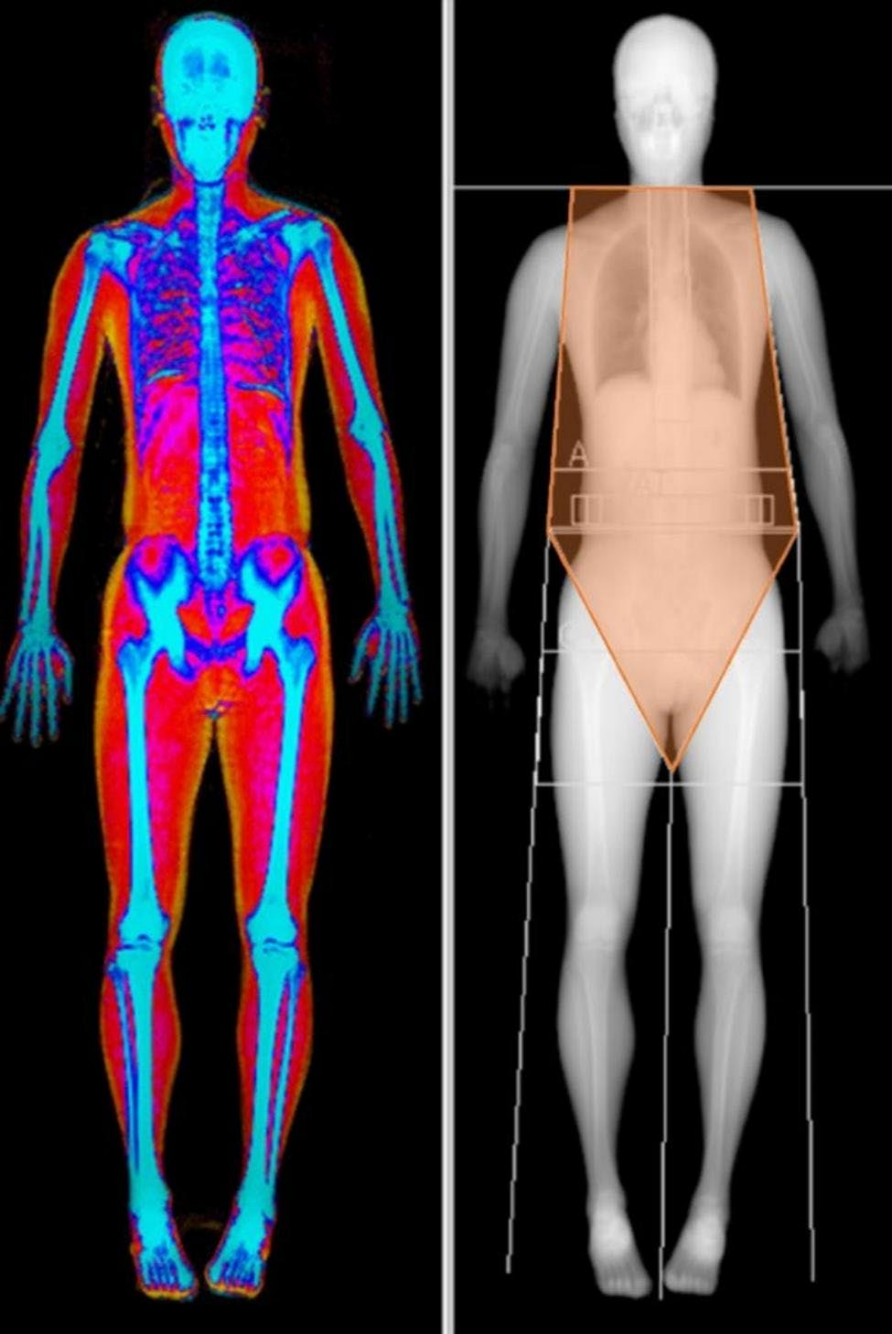
Dual-energy X-ray absorptiometry (DXA) scan with lean mass analysis Colors in the DXA images represent tissue densities, with yellow (fat) being the lowest density and blue (bone) being the highest density. Trunk muscle mass is defined as the trunk fat-free mass, excluding the head and all limbs

DXA (Hologic Horizon W, Waltham, MA, USA) was performed on the whole body for all participants. Trained technicians performed the DXA, which were calibrated every morning. DXA uses a source that generates X-rays, a detector, and an interface with a computer system for imaging the scanned areas of interest and provides an estimate of three body compartments: lean, bone, and fat. At bone locations, lean and soft tissue are interpolated from the surroundings. These measurements can be performed for whole body and for several regions (e.g., trunk, arms, and legs). Trunk muscle mass (TMM) was determined thorough the value of trunk lean mass which is lean mass in the body regions excluding head and all appendicular (upper and lower limbs) regions from body composition data measured using DXA (Fig. 1). We used the same TMM values obtained from DXA to correlate the CSA of each musculature and did not change the region of the TMM according to the level of muscle CSA.

### MRI

**Fig. 2:**
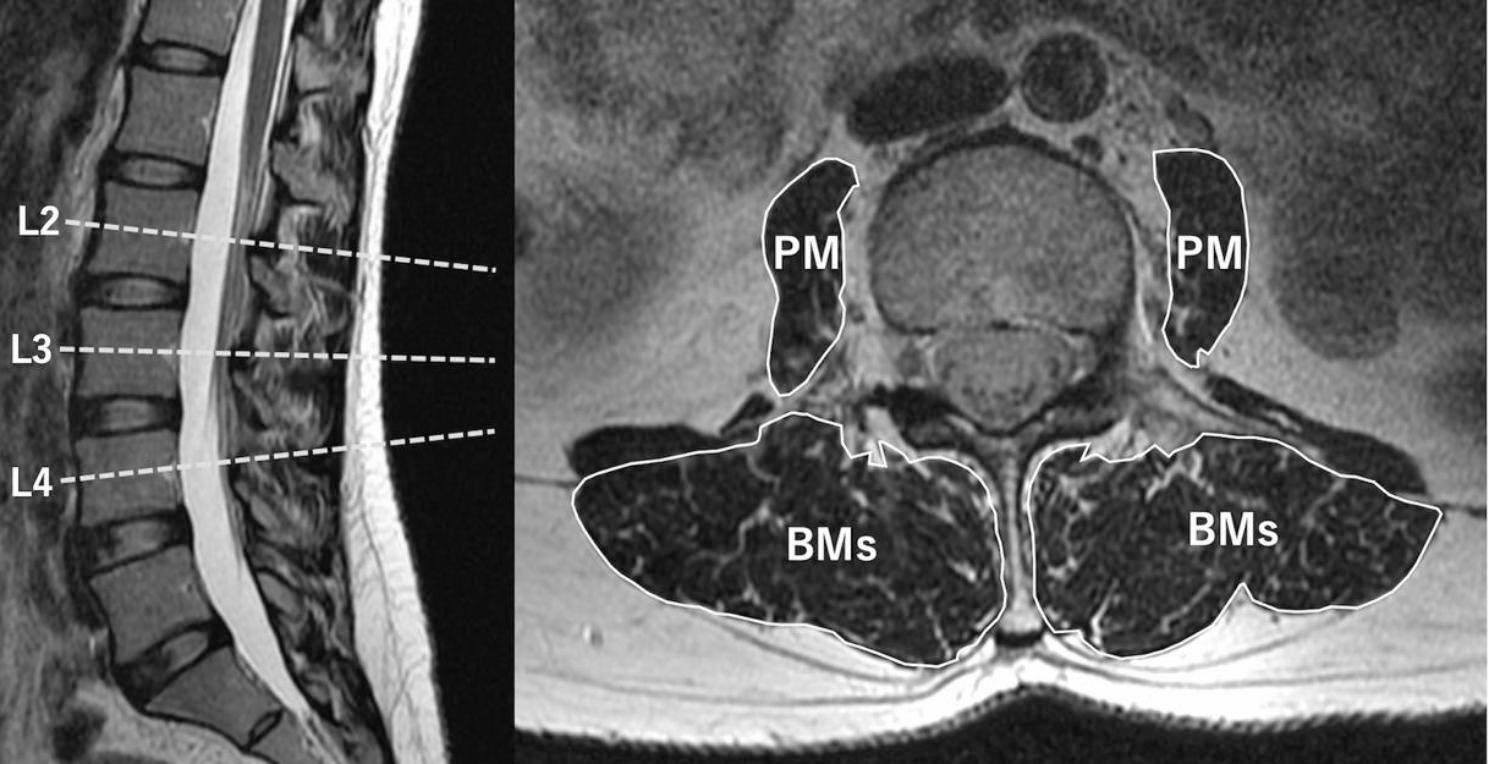
Cross-sectional areas (CSA) of the psoas major (PM) and back muscles (BMs) CSA of the PM and BMs were obtained bilaterally in the middle of L2, L3, and L4 on T2-weighted lumbar axial MR images

MRI (Signa HDxt 1.5T GE Healthcare, Waukesha, WI, USA) was performed on all participants on the same day as DXA. Trained technicians performed the MRI, which were calibrated every morning. During imaging, the participants were supine while maintaining a neutral spine position with a pillow placed under their knees. CSA of the psoas major and back muscles was measured on T2-weighted axial MR images (repetition time/echo time = 1625–4000 ms/ 84–88 ms) of the lumbar spine. Some authors have asserted that the CSA of the paraspinal muscles is at or near maximal at the upper endplate of L4 [19, 20], whereas others have found that the CSA of the back muscles is maximal at approximately L3 [21, 22]. In this study, three levels were analyzed to obtain the most accurate and suitable muscular CSA. The measured levels were in the middle of the L2, L3, and L4 levels (Fig. 2).

**Fig. 3:**
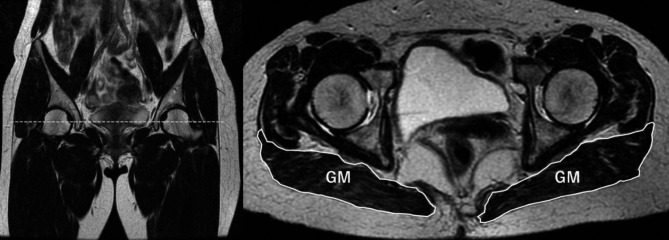
Cross-sectional areas (CSA) of the gluteus maximus (GM) CSA of the GM were obtained bilaterally at the center of the femoral head on a T2-weighted hip axial MR image

Since TMM measured using DXA included spine and pelvis regions, the gluteus maximus was also evaluated on T2-weighted axial MR images (repetition time/echo time = 3085–6572 ms/ 101–104 ms) of the pelvis. The measured level was at the center of the femoral head (Fig. 3).

The regions of interest were defined by manually tracing the fascial boundaries of each muscle on both sides. The regions of interest were analyzed using a digitalized image processing software (Image J, National Institutes of Health, Bethesda, MD, USA). The value of each muscular CSA was calculated as the sum of the CSA on both sides.

Correlation and linear regression between DXA-derived TMM and the sum of the CSA of the psoas major on L2, L3, and L4; the correlation and linear regression between DXA-derived TMM and the sum of the CSA of the back muscles on L2, L3, and L4; and the correlation and linear regression between DXA-derived TMM and the CSA of the gluteus maximus on the femoral head were calculated separately for men and women.

### Statistical analyses

Statistical analyses were performed using JMP Pro version 16.0 statistical software (SAS Institute, NC, USA). Correlations between TMM measured using DXA and CSA of each musculature were analyzed using Spearman’s rank correlation coefficient. Pearson’s correlation coefficient is affected when the distribution contains extremely large or small values, such as outliers. Because the number of subjects was not large, Spearman’s correlation coefficient was used [23]. Correlation strength was categorized as very week (< 0.20), weak (0.20–0.39), moderate (0.40–0.59), strong (0.60–0.79), or very strong (≥ 0.80). Linear regression analysis between the TMM and CSA of each musculature was performed to create equations. *P*-values < 0.01 were considered statistically significant.

## Results

A total of 48 healthy volunteers participated in this study. The study included 23 men and 25 women, with a mean age of 47 (28–68) years. The demographic data included age, sex, height, weight, body mass index, skeletal muscle mass index, the ODI and RDQ (Table 1).

**Table 1:** Baseline characteristics of participants

	Men (n = 23)	Women (n = 25)
Age	47 ± 13	47 ± 11
Height (cm)	170 ± 6.4	158 ± 5.9
Weight (kg)	69.0 ± 8.7	57.8 ± 9.6
BMI (kg/m^2^)	24.0 ± 2.9	23.1 ± 3.5
SMI (kg/m^2^)	7.6 ± 1.1	5.7 ± 0.8
ODI (%)	1.5 ± 3.3	3.7 ± 4.2
RDQ	0.1 ± 0.3	0.6 ± 1.6

Abbreviations: BMI body mass index, SMI skeletal muscle mass index, ODI Oswestry Disability Index, RDQ Roland-Morris Disability Questionnaire

DXA-derived TMM was 23.8 kg in men, and 18.0 kg in women. Sum of CSA of psoas major on L2, L3 and L4 was 6767 mm^2^ in men, and 3441 mm^2^ in women. Sum of CSA of back muscles on L2, L3 and L4 was 14,820 mm^2^ in men, and 10,941 mm^2^ in women. CSA of gluteus maximus was 10,190 mm^2^ in men, and 7947 mm^2^ in women (Table 2).

**Table 2:** Trunk muscle mass (TMM) measured using dual-energy X-ray absorptiometry (DXA) and cross-sectional areas (CSA) of each musculature measured using magnetic resonance imaging

		Men (n = 23)	Women (n = 25)
DXA-derived TMM (kg)		23.8 ± 3.0	18.0 ± 2.3
CSA of psoas major (mm^2^)	L2	1330 ± 459	665 ± 201
	L3	2285 ± 591	1132 ± 270
	L4	3153 ± 698	1645 ± 415
	Sum of L2-4	6767 ± 1672	3441 ± 808
CSA of back muscles (mm^2^)	L2	4856 ± 712	3267 ± 537
	L3	5066 ± 695	3612 ± 569
	L4	4898 ± 656	4061 ± 484
	Sum of L2-4	14,820 ± 1912	10,941 ± 1487
CSA of gluteus maximus (mm^2^)		10,190 ± 1627	7947 ± 1411

Abbreviations: DXA dual-energy X-ray absorptiometry, TMM trunk muscle mass, CSA cross-sectional areas

**Fig. 4:**
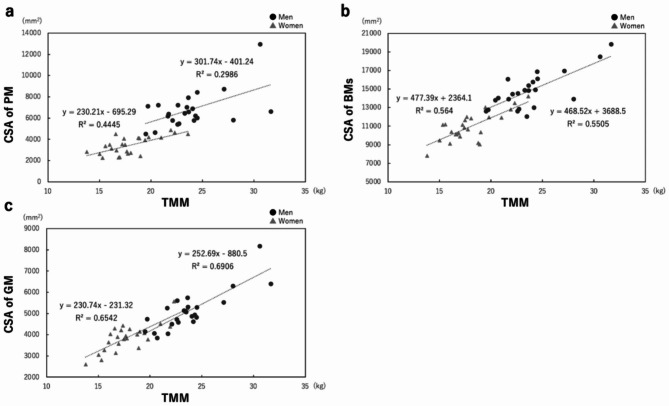
Correlations between trunk muscle mass (TMM) measured using dual-energy X-ray absorptiometry (DXA) and cross-sectional areas (CSA) of each musculature measured using magnetic resonance imaging Correlation between DXA-derived TMM and CSA of the psoas major (PM) (**a**). Correlation between DXA-derived TMM and CSA of the back muscles (BMs) (**b**). Correlation between DXA-derived TMM and CSA of the gluteus maximus (GM) (**c**)

There was a weak correlation between the TMM and CSA of the psoas major in men (r = 0.39, *P* = 0.0678) and a moderate correlation between the TMM and CSA of the psoas major in women (r = 0.58, *P* = 0.0021). Moderate correlations were observed between TMM and CSA of the back muscles in both men (r = 0.63, *P* = 0.0012) and women (r = 0.63, *P* = 0.0007). There was a strong correlation between the TMM and CSA of the gluteus maximus in men (r = 0.72, *P* < 0.0001) and a moderate correlation between the TMM and CSA of the gluteus maximus in women (r = 0.69, *P* < 0.0001). The linear regression equation between the TMM and CSA of the psoas major was y = 301.74x – 401.24 in men (R^2^ = 0.2976, *P* = 0.0070) and y = 230.21x − 695.29 in women (R^2^ = 0.4445, *P* = 0.0003). The linear regression equation between the TMM and CSA of the back muscles was y = 468.52x + 3688.5 in men (R^2^ = 0.5505, *P* < 0.0001) and y = 477.39x + 2364.1 in women (R^2^ = 0.564, *P* < 0.0001). The linear regression equation between the TMM and CSA of the gluteus maximus was y = 252.69x − 880.5 in men (R^2^ = 0.6906, *P* < 0.0001) and y = 230.74x – 231.32 in women (R^2^ = 0.6542, *P* < 0.0001) (Fig. 4).

## Discussion

Several techniques, including MRI, CT, DXA, and BIA, are available for measuring muscle size [24]. MRI and CT have high validity, but are complex and costly. DXA and BIA are simple and inexpensive, and more realistic clinical methods. Previous studies have shown that DXA is low cost, has low radiation exposure (< 1 μSv for whole-body scans), and is reliable for research setting [9, 25, 26].

Although all linear regression equations between the DXA-derived TMM and CSA of the psoas major, back muscles, and gluteus maximus were significant, the correlation between the DXA-derived TMM and CSA of the psoas major was lower than that in the other musculature. This may be because the iliopsoas muscle is not used in forward bending except to initiate forward bending, since hip flexion passively causes a backward tilt of the pelvis. However, finding a significant correlation between DXA-derived TMM and CSA of the back muscles, which is closely related to spinal disorders, is very important.

DXA-derived TMM was strongly correlated with the CSA of the gluteus maximus. For many years, it has been shown that bending forward is a two-part movement that involves both the spine and pelvis. In extension from the fully flexed position, the movement is reversed so that trunk extension is achieved through cooperative contraction of the hip extensors, including the gluteus maximus and muscles of the back [27, 28]. These findings suggest that the gluteus maximus plays an important role in maintaining sagittal spinal alignment and is a key muscle in the hip extensor. Bao et al. also reported a close relationship between the gluteal muscles and sagittal malalignment [29]. Because DXA-derived TMM is an indicator of gluteus maximus size, longitudinal measurements of TMM may help in the early detection and prevention of spinal disorders with sagittal malalignment.

The current study has several limitations. The participants were young and middle-aged healthy volunteers. Our findings in this study may not be generalizable to other populations, such as older adults. Second, the possibility of self-report bias in low back pain cannot be eliminated, but questionnaires (ODI and RDQ) used to ensure that participants did not have function-limiting low back pain have been found to be reliable and valid decreasing this possibility. Finally, there is a belief that muscle strength and endurance are clinically more important than muscle size, and that muscle size may not be a major predictor of muscle strength and physical performance. Wang et al. reported that muscle density may represent a more clinically meaningful surrogate of muscle performance than muscle size [30]. Future studies should focus on analyzing the quality of muscle as well as quantity of muscle.

In this study, we investigated the values of DXA-derived TMM and muscle CSA measured using MRI in healthy subjects. In the future, we would like to investigate patients with lumbar degenerative disease and spinal deformities.

## Conclusions

The DXA-derived TMM was able to predict the CSA of the psoas major, back muscles, and gluteus maximus, and significantly correlated with the CSA of the back muscles and gluteus maximus in healthy volunteers regardless of sex. Whole-body DXA might be a safer and cheaper alternative for evaluating the size of the back muscles and gluteus maximus.

## Data Availability

All data supporting our findings are contained within the manuscript.

## Abbreviations

BIA: Bioelectrical impedance analysis
BMI: Body mass index
BMs: Back muscles
CSA: Cross-sectional areas
DXA: Dual-energy X-ray absorptiometry
GM: Gluteus maximus
ODI: Oswestry Disability Index
PM: Psoas major
RDQ: Roland-Morris Disability Questionnaire
SMI: Skeletal muscle mass index
TMM: Trunk muscle mass
